# Intestinal mucosal barrier: a potential target for traditional Chinese medicine in the treatment of cardiovascular diseases

**DOI:** 10.3389/fphar.2024.1372766

**Published:** 2024-02-26

**Authors:** Jiahui Liu, Xiunan Wei, Tong Wang, Miaomiao Zhang, Ying Gao, Yan Cheng, Lili Chi

**Affiliations:** ^1^ College of First Clinical Medicine, Shandong University of Traditional Chinese Medicine, Jinan, China; ^2^ College of Nursing, Shandong University of Traditional Chinese Medicine, Jinan, China; ^3^ Affiliated Hospital of Shandong University of Traditional Chinese Medicine, Jinan, China

**Keywords:** metabolites of Chinese botanical drugs, traditional Chinese medicine formulas, Chinese patent medicine, cardiovascular disease, intestinal mucosal barrier, gut microbiota

## Abstract

Cardiovascular disease (CVD) is a serious public health problem, and among non-communicable diseases, CVD is now the leading cause of mortality and morbidity worldwide. CVD involves multiple organs throughout the body, especially the intestinal tract is the first to be involved. The impairment of the intestinal mucosal barrier is considered a significant pathological alteration in CVD and also contributes to the accelerated progression of the disease, thereby offering novel insights for CVD prevention and treatment. The treatment of Chinese medicine is characterized by multi-metabolites, multi-pathways, and multi-targets. In recent years, the studies of Traditional Chinese Medicine (TCM) in treating CVD by repairing the intestinal mucosal barrier have gradually increased, showing great therapeutic potential. This review summarizes the studies related to the treatment of CVD by TCM (metabolites of Chinese botanical drugs, TCM formulas, and Chinese patent medicine) targeting the repair of the intestinal mucosal barrier, as well as the potential mechanisms. We have observed that TCM exerts regulatory effects on the structure and metabolites of gut microbiota, enhances intestinal tight junctions, improves intestinal dyskinesia, repairs intestinal tissue morphology, and preserves the integrity of the intestinal vascular barrier through its anti-inflammatory, antioxidant, and anti-apoptotic properties. These multifaceted attributes position TCM as a pivotal modulator of inhibiting myocardial fibrosis, and hypertrophy, and promoting vascular repairment. Moreover, there exists a close association between cardiovascular risk factors such as hyperlipidemia, obesity, and diabetes mellitus with CVD. We also explore the mechanisms through which Chinese botanical drugs impact the intestinal mucosal barrier and regulate glucose and lipid metabolism. Consequently, these findings present novel insights and methodologies for treating CVD.

## Graphical Abstract

**Figure:**
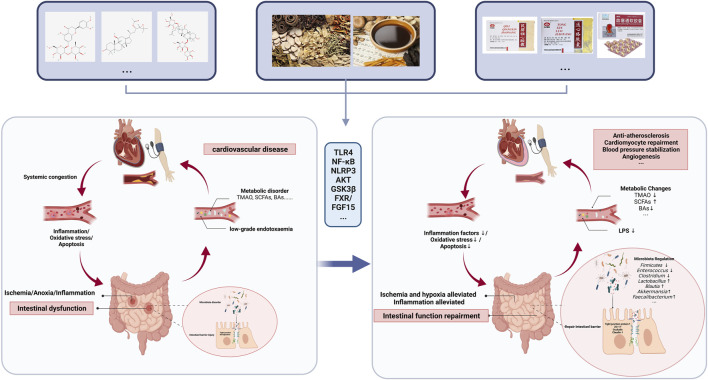
This review presents the progress of traditional Chinese medicines in treating CVD by repairing the intestinal mucosal barrier

## Introduction

CVD poses a significant global health threat, affecting over 23 million individuals worldwide (Bui et al., 2011). In China, the incidence of CVD is steadily increasing each year, making it the leading cause of hospitalization and contributing to the overall disease burden (Wang et al., 2021a; Ren et al., 2021). CVD is a systemic ailment that impacts multiple organ systems beyond just the cardiovascular system, including the intestinal tract which often experiences initial manifestations (Krack et al., 2005; Keeter et al., 2022). Medications used for treating CVD such as nonsteroidal anti-inflammatory drugs (NSAIDs) and antithrombotic drugs can potentially compromise the integrity of the gastrointestinal mucosal barrier and lead to drug-induced gastrointestinal ulcers (US Preventive Services Task Force et al., 2022). Severe mucosal damage may result in intestinal dysfunction or even failure (Arutyunov et al., 2008; Krack et al., 2005). Experimental studies have demonstrated that impairment of the intestinal mucosal barrier, particularly alterations in gut microbiota and its metabolites, are closely associated with pathological mechanisms like inflammation, ischemia, and hypoxia, as well as metabolic disorders (Wang and Zhao, 2018). Targeting intestinal barrier dysfunction could potentially serve as a therapeutic approach for managing CVD (Lewis and Taylor, 2020).

However, drug studies targeting the intestinal mucosal barrier for CVD treatment in medicine are scattered across clinical and experimental reports. Recent research has demonstrated that candesartan cilexetil, a renin-angiotensin system blocker, not only reduces blood pressure and improves cardiac function but also repairs damage to the intestinal barrier (Wu et al., 2019a). Lubiprostone, a ClC-2 chloride channel activator utilized for constipation treatment, has demonstrated efficacy in reducing the lactulose/mannitol ratio in patients with NSAID-induced intestinal barrier dysfunction (Kato et al., 2017). Additionally, it has been found to restore the intestinal barrier and ameliorate inflammation in Western diet-induced Apolipoprotein E-deficient (ApoE−/−) mice by attenuating lipids, thereby mitigating atherosclerosis (AS) (Arakawa et al., 2019). Probiotics and prebiotics have demonstrated efficacy in clinical trials (Jin et al., 2019). However, the research and development of these therapeutic agents are still ongoing. The application of TCM formulas for CVD is extensively utilized in clinical settings (Chen et, al., 2023a). Hao et al. (Hao et al., 2017) systematically evaluated the efficacy and safety of TCM for CVD in conjunction with the potential pharmacological mechanisms of the active ingredients. The results suggest that TCM can be used as a complementary and alternative approach to the prevention and treatment of CVD. Many botanical drugs and their metabolites have been shown to repair intestinal barrier damage and improve the intestinal microbiota in CVD therapy (Li et al., 2017; Lyu et al., 2017). This article discusses the potential mechanisms by which botanical metabolites, botanical drugs, and proprietary Chinese medicines act on CVD from the perspective of the intestinal mucosal barrier and gut microbiota.

## Relationship between the intestinal mucosal barrier and CVD

### Functions of the intestinal mucosal barrier

The intestinal tract is the largest immune organ in the human body, and the intestinal mucosal barrier is an extremely important part of the intestinal tract that prevents the invasion of pathogens (Camilleri, 2019). Digestive juices and commensal bacteria in the gut degrade bacteria and antigens. Paneth cells secrete antimicrobial peptides that prevent bacterial colonization (Wang et al., 2018), and cuprocytes secrete a mucin barrier that prevents pathogen attachment (Johansson and Hansson, 2016). Tight junction proteins, including the occludin, claudin, zonula occludens, and junctional adhesion molecule families, prevent pathogen translocation (Odenwald and Turner, 2017). The lamina propria secretes IgA, cytokines, chemokines, and immune cells to play an immune role (Johansson and Hansson, 2016; Camilleri, 2019). In addition, the gut vascular barrier (GVB) is the last line of defense of the intestinal mucosal barrier and recognizes translocated microorganisms and antigens in the bloodstream (Spadoni et al., 2015).

### Intestinal dysfunction in cardiovascular disease

Cardiovascular systems, such as heart, cardiac macro vessels, and capillaries are related to the circulation of the entire organism (Rakusan et al., 1992), and the gastrointestinal system contains abundant blood vessels, receiving about ¼ of the blood supply of the heart and is sensitive to ischemia and hypoxia (Krack et al., 2005). The presence of systemic inflammation and inadequate perfusion caused by cardiovascular disease results in a reduction in intestinal peristalsis, absorption, and the development of edema in the intestinal wall. This leads to disruption of the mucus layer, disarray within the bacterial flora, and loss of tight junctions, ultimately resulting in increased permeability commonly referred to as “leaky gut” (Usuda et al., 2021). Sandek et al. (2014) found that intestinal blood flow is reduced, and gastrointestinal symptoms are increased in patients with chronic heart failure (CHF). The study conducted by Drapala et al. (2020) demonstrated that intestinal blood barrier disturbances, such as decreased intestinal blood flow, reduced colonic mucosal thickness, and alterations in tight junctions, were observed in spontaneously hypertensive HF rats. Chronic inflammatory states induced by AS and dyslipidemia are involved in vascular injury and microvascular remodeling, increasing intestinal vascular permeability (Witjes et al., 2015). The combination of congestive heart failure, atrial fibrillation (AF), and other factors leads to intestinal hypoperfusion, while a hypercoagulable state of blood and blood stasis contribute to the development of intestinal ischemia (Mosinska and Fichna, 2015; Wang et al., 2016). Thus, both CVD itself and CVD-related risk factors (obesity, high-fat diet, diabetes, etc.) affect the function and structure of the intestinal barrier (Zhang et al., 2020a; Ding et al., 2020). The internal environment, such as inflammation and hypoxia, plays an important role (Kalogeris et al., 2016; Yuzefpolskaya et al., 2020). In a rat model of myocardial infarction, serum and intestinal levels of TNF-α and IL-6 were significantly increased, and IL-10 levels were significantly decreased, accompanied by increased tissue levels of cyclooxygenase-2 (COX-2) and positively correlated with matrix metalloproteinase-2 (MMP-2) activity (Chen et al., 2021).

### Intestinal permeability and cardiovascular risk

After leaky gut, pathogens in the intestine as well as pathogen-associated molecular patterns (PAMPs), such as the harmful metabolite lipopolysaccharide (LPS), can readily leak from the damaged barrier into the bloodstream, causing systemic disease (Tripathi et al., 2018). Among them, LPS, a major component of the outer membrane of Gram-negative bacteria, translocates into the systemic circulation to develop low-grade endotoxaemia (Violi et al., 2023). Low-grade endotoxemia induces arterial inflammation, plaque instability, and thrombosis, which may lead to atherosclerosis and has been associated with cardiovascular events in patients at risk for or suffering from serious cardiovascular disease associated with cardiovascular events in patients at risk or with severe cardiovascular disease (Violi et al., 2023). Serum LPS levels are elevated in STEMI patients and correlate with levels of zonulin, a marker of intestinal permeability, and LPS is localized in coronary thrombi in STEMI patients (Carnevale et al., 2020). Zhang et al. (2022) found that intestinal barrier dysfunction in rats with a dramatic increase in LPS and glucose levels led to upregulated expression of NOD-like receptor protein-3 (NLRP-3) inflammasome, which promoted the development of AF. This may be one of the important reasons why intestinal disease induces the development of CVD and accelerates the development of CVD.

### The role of gut microbiota in CVD

Gut microbiota is a very important part of the intestinal barrier, and intestinal commensal bacteria maintain intestinal homeostasis (Wells et al., 2017). The gut microbiota influences host immunity, metabolism, and development (Rosell-Mases et al., 2023). Structural disruption of gut microbiota or small intestinal bacterial overgrowth (SIBO) leads to impairment of intestinal barrier function, which directly or indirectly causes diseases, including CVD (Brown and Hazen, 2015; Adkins and Rezaie, 2018). Zhang et al. (2022) found that fecal Firmicutes transplantation (FMT) of feces from aged rats with high susceptibility to AF to young healthy rats increased AF susceptibility in young rats, whereas FMT of long-term healthy young rats to older rats prevented AF in older rats. Increased Firmicutes to Bacteroidetes ratios were found in the feces of hypertensive models and patients, whereas Coprococcus Pseudobutyrivibrio and Bifidobacterium, which produce acetate, butyrate, and lactate, were decreased (Yang et al., 2015).

The gut microbiota generally affects CVD through two pathways. One way is through changes in metabolites such as trimethylamine N-oxide (TMAO), bile acids (BAs), short-chain fatty acids (SCFAs), and tryptophan derivatives (Liu et al., 2021a). The gut microbiota can convert various dietary nutrients into trimethylamine (TMA). Most TMA enters the circulation and is subsequently oxidized to TMAO by heparin-containing monooxygenase (FMO) (Bennett et al., 2013). Studies have shown that TMAO is associated with strong pro-AS activity and myocardial remodeling, leading to a higher risk of adverse cardiovascular events (Tang et al., 2013; Organ et al., 2016; Zhu et al., 2016). The gut microbiota modifies primary BA through products such as 7α-dehydroxylase, and bile salt hydrolase (BSH), to produce secondary BA (Walker and Gilliland, 1993; Ridlon et al., 2006), which is associated with farnesoid X receptor (FXR), liver X receptor (LXR), pregnancy X receptor (PXR), and G protein-coupled receptor (GPCR) and interacts with various host nuclear receptors, causing lipid metabolism disorders and cardiovascular metabolic diseases such as AS (Hanniman et al., 2005; Hylemon et al., 2009; Pols et al., 2011; Li et al., 2013).SCFA is recognized as a beneficial factor for CVD (Hu et al., 2022). Cohort studies have shown that changes in fecal SCFAs, including acetate, propionate, and butyrate, are significantly associated with 24-h mean blood pressure in hypertensive patients (Huart et al., 2021). Intestinal butyrate-producing bacteria are enriched in the intestines of patients with STSMI infarction and rhesus monkeys undergoing ischemia-reperfusion, and supplementation with butyrate and colonization with butyrate-producing bacteria improves cardiac function and myocardial injury after myocardial injury (Chen et al., 2023a). Propionate was found to attenuate myocardial hypertrophy, myocardial fibrosis, vascular dysfunction, and hypertension in Ang II-induced wild-type NMRI mice or ApoE−/− mice (Bartolomaeus et al., 2019).

Another way is for the gut microbiota to induce changes in the immune pathways of the host. LPS can be sensed by Toll-like receptor 4 (TLR-4), which activates macrophages to take up oxidized low-density lipoproteins (ox-LDL) to form foam cells, triggering atherosclerotic progression (Caesar et al., 2010). A reduction in the relative abundance of butyrate-producing Roseburia and Faecalibacterium, as well as an increase in the TNFα: IFN-γ ratio and the production of TNFα and IL-6 in isolated peripheral blood mononuclear cells, can be observed in hypertensive patients (Kim et al., 2014). Butyrate has also been found to modulate CD4^+^ T helper cell differentiation, exerting an anti-inflammatory effect and thus influencing CVD (Hu et al., 2022).

## Potential mechanisms of metabolites of Chinese botanical drugs affecting intestinal mucosal barrier for the treatment of CVD

### Phenols

Resveratrol (RSV), is a plant polyphenol phytoalexin (Baur and Sinclair, 2006), which is mainly found in Chinese botanical drugs such as Smilax glabra Roxb. (Smilacaceae; Smilacis glabrae rhizoma), Veratrum album L. (Melanthiaceae; Veratrum album) and other traditional Chinese medicines. RSV could decrease TMAO levels and increase the abundance of BSH-active bacteria such as *Lactobacillus* and Bifidobacterium, which reduces BA degradation and fecal excretion. In contrast, RSV was found to neither reduce TMAO levels nor increase hepatic BA synthesis in antibiotic-treated sham-naive mice, suggesting that RSV acts by remodeling the gut microbiota. The FXR/FGF15 axis was inhibited in this process, and cholesterol 7a-hydroxylase (CYP7A1) expression was increased, exerting an anti-AS effect (Chen et al., 2016). This suggests that the beneficial cardiovascular effects of RV are related to the composition of the gut microbiota. According to Chen et al., (Chen et al., 2019), RV also prevents programmed hypertension associated with an increased abundance of the abundances of phylum Verrucomicrobia and genus Akkermansia Muciniphila (Chen et al., 2019).

Quercetin, a plant-derived polyphenol, found in Glehnia littoralis (A.Gray) F.Schmidt ex Miq. (Apiaceae; glehniae radix), Eucommia ulmoides Oliv. (Eucommiaceae; Eucommiae cortex) and many other botanical drugs. Quercetin treatment effectively attenuated weight gain and mitigated the extent of atherosclerotic lesions in the aortic sinus. The observed reduction in malondialdehyde levels and elevation of IL-6 levels further substantiated the protective effects of quercetin on immune/inflammatory responses and oxidative stress (Nie et al., 2019). Moreover, quercetin supplementation led to decreased intestinal cholesterol, lysophosphatidic acids, and atherogenic lysophosphatidylcholine (LPC 18:1) levels, while promoting an increased level of coprostanol (Nie et al., 2019). Microbiological analyses unveiled that quercetin treatment significantly enhanced Actinobacteria and Bacteroidetes populations, whereas it markedly reduced Firmicutes abundance (Nie et al., 2019).

Baicalin is found in botanical drugs, such as Scutellaria baicalensis Georgi (Lamiaceae; Scutellariae radixp). Wu et al. found in SHRs that baicalin attenuated hypertension-associated intestinal hyperpermeability and reduced serum high-sensitivity C-reactive protein (hs-CRP), inflammatory markers such as IL-1β and IL-6 (Wu et al., 2019a). And Baicalin treatment increased the abundance of SCFA-producing fecal flora, such as *Streptococcus*, Akkermansia, Allobaculum, Bifidobacterium, Lachnospiraceae_NK4B4_group, and Roseburia, as well as increased levels of SCFA (Wu et al., 2019b).

Naringin is a flavanone-7-O-glycoside between the flavanone Naringenin and the disaccharide neohesperidose. Naringin could promote the growth of 7α-dehydroxylase-producing bacteria, such as Eubacterium coprostanoligenes and Eubacterium brachy, and inhibit BSH-producing bacteria, such as *Clostridium*, *Enterococcus*, and *Bacteroides*, which may be important in alleviating AS by increasing bile acid synthesis through activation of the FXR/FXR/Fibroblast Growth Factor 15 (FGF15)-Cytochrome P450 7A1 (CYP7A1) pathway, thereby lowering cholesterol levels (Wang et, al., 2021a). Furthermore, the integrity of the GVB represents the ultimate defense mechanism for controlling bacterial translocation, with particular emphasis on the pivotal role played by the vascular endothelium within this context (Spadoni et al., 2016). The TNF-α-induced injury model of rat intestinal microvascular endothelial cells (RIMVEC) serves as a valuable tool to mimic inflammation-induced damage to GVB. Notably, intervention with Naringin effectively ameliorated tight junction proteins in RIMVEC, including Zonula Occludens-1 (ZO-1), occludin, and claudin-1, thereby preventing apoptosis and inhibiting cell migration while mitigating GVB disruption (Liu et al., 2020).

Naringenin is found in botanical drugs, such as Pericarpium Citri Reticulatae (Rutaceae; Citrus reticulata Blanco), and Paeonia lactiflora Pall. (Paeoniaceae; Paeoniae Radix Rubra). Naringenin has also been demonstrated to effectively mitigate TNF-α-induced disruption of the RIMVECs, as evidenced by the restoration of occludin and claudin-1 expression levels. This effect is partly attributed to the inhibition of nuclear factor kappa-B (NF-κB)-mediated activation of the Myosin Light Chain Kinases (MLCK)/p-MLC and TLR4/NF-κB/NLRP3 pathways (Zhong et al., 2021). Intestinal barrier dysfunction, including gastrointestinal motility disturbances, is associated with aberrant smooth muscle function and structure. Naringenin has been shown to hyperpolarize colonic smooth muscle cells (SMCs) through selective activation of large-conductance calcium-activated K+ (BKCa) channels, thereby reducing Ca2+ influx via voltage-dependent calcium channels (VDCC) and promoting SMCs relaxation for treating dyskinesia in rat colon (Yang et al., 2014). The activating effect of Naringenin on BKCa channels is also observed in vascular smooth muscle. In rat tail artery myocytes, (+/−)-Naringenin concentration-dependently increased BKCa currents. Additionally, (+/−)-Naringenin exerted a concentration-dependent relaxing effect on endothelium-denuded rat aortic rings precontracted with 20 mM KCl or norepinephrine (Saponara et, al., 2006). Naringenin not only regulates gastrointestinal motility disorders, but also exerts vasodilatory effects, but is not found in the same model, and its intrinsic mechanism of action needs to be investigated.

### Terpenoids

Notoginsenoside R1 (NGR1) is a metabolite from Panax notoginseng (Burkill) F.H.Chen (Araliaceae; Notoginseng radix et rhizoma)NGR1 significantly reduced myocardial infarct size, alleviated cardiomyocyte injury, decreased cardiomyocyte apoptosis, and improved cardiac function in mice with myocardial ischemia/reperfusion (MI/R) by inhibiting transforming growth factor β-activated protein kinase 1 (TAK1)/c-Jun amino-terminal kinase (JNK). Pretreatment with NG-R1 (25 μM) significantly inhibited apoptosis in murine neonatal cardiomyocytes (CMs) induced by hypoxia/reoxygenation (H/R) (Zeng et al., 2023). Low-grade endotoxemia, resulting from increased intestinal permeability, stimulates leukocytes, platelets, and endothelial cells. This leads to inflammation and a hypercoagulable state, which in turn causes plaque instability and thrombosis (Violi et al., 2023). NGR1 inhibited the LPS-induced degradation of IκB-α (inhibitor of NF-κB) and increased synthesis of IκB-α protein in monocytic cell line THP-1. Simultaneously, it prevented LPS-induced upregulation of plasminogen activator inhibitor-1 (PAI-1) antigen and tissue factor in endothelial cells, improved LPS-induced PAI-1 in mice, and exerted anti-inflammatory and anti-thrombotic effects (Zhang et al., 1997). Interestingly, NGR1 also plays a protective role in intestinal ischemia-reperfusion (I/R). NGR1 attenuated intestinal I/R-induced microvascular hyperpermeability, reduced inflammatory cytokine production such as jejunal TNF-α, IL-1β, and IL-6, activated the NF-κB pathway and increased tight junction proteins, and improved energy metabolism during intestinal I/R (Li et al., 2014).

Ginsenoside Rc (GRc) is Panax ginseng C.A.Mey. (Araliaceae; Ginseng radix et rhizoma), and many drugs for the treatment of CVD contain ginsenoside, such as Shexiang Baoxin Pill, Tongxinluo, etc. Xie et al. (2022) found that GRc significantly attenuated AS injury in high-fat diet (HFD)-fed ApoE−/− mice by lowering blood lipids, IL-6, and IL-1β serum levels. And they also found that GRc reversed AS-induced changes in the abundance of Bacteroidetes and Firmicutes, Muribaculaceae, *Lactobacillus*, Ileibacterium, Bifidobacterium, Faecalibaculum, Oscillibacter, Blautia, and Eubacterium_Coprostanoligenes_group and other flora (Xie et al., 2022). Fecal metabolomics revealed that GRc treatment significantly reduced adenine and uric acid and was positively correlated with serum levels of TG, IL-6, IL-1β, and TNF-α (Xie et al., 2022). Oxidative stress, vascular injury, and systemic inflammation due to elevated uric acid have deleterious effects on CVD (Ndrepepa, 2018). GRc treatment also elevates levels of cholic acid (CA), chenodeoxycholic acid (CDCA), isolithocholic acid (isoLCA), taurochenodeoxycholic acid (TCDCCA), and tricarboxylic acid (TCA) (Xie et al., 2022). Primary bile acids, such as CA and CDCA, as well as secondary bile acids can activate FXR and TGR5 and reduce AS formation (Miyazaki-Anzai et al., 2018; Schoeler and Caesar, 2019). This may be one of the mechanisms by which GRc mitigates AS damage.

### Alkaloids

Berberine (BBR) was revealed to inhibit deoxynivalenol-induced intestinal injury by increasing the expression of serum antioxidant enzymes and T-cell surface antigens and by decreasing the release of pro-inflammatory cytokines in the small intestine. BBR significantly increased ileal and jejunal mucosal ZO-1 by decreasing the expression of deoxynivalenol (DON)-induced genes and proteins in the jejunum and ileum in the presence of extracellular regulated protein kinases (ERK), JNK, and NF-κB, occludin and claudin-1 protein expression levels, and improved jejunal morphology. BBR is also known to improve jejunal morphology (Tang et, al., 2021). BBR treatment significantly reduced AS area and lipid levels decreased pro-inflammatory cytokines TNF-α, IL-1β, IL-6, and increased anti-inflammatory IL-10 and lipocalin levels in HFD-fed ApoE−/− mice (Wu et al., 2020). In this study, 16S rRNA sequencing and macrogenic findings revealed that BBR altered the abundance of Roseburia, Blautia, Allobaculum, Alistipes, Turicibacter, and Bilophila. These colonies showed beneficial anti-inflammatory effects, modulation of glycolipid metabolism, increased SCFAs, and decreased TMAO potential (Wu et al., 2020). Furthermore, BBR has been utilized for treating atherosclerotic patients (0.5 g, bid). However, oral administration of 5 g bid of BBR for 4 months resulted in an increased susceptibility to plaque formation among these patients. Notably, there was a significant decrease observed in the abundance of TMA-producing potential gut microbiota species, including Eubacterium_hallii_group, Anaerostipes, Faecalibacterium, Dialister, Eubacterium_coprostanoligenes_group, Coprococcus_3, Butyricoccus, and *Clostridium*_sensu_strito_1. Additionally, fecal and stool levels of both TMA and TMAO were significantly reduced along with a notable decrease in plaque score (Ma et al., 2022). Another study showed that BBR protects against NSAID-induced intestinal mucosal damage by upregulating the expression of Protein gene product 9.5 (PGP9.5), glial fibrillary acidic protein (GFAP), and Glial-cell-line-derived neurotrophic factor (GDNF) with the repair of enteric nervous system (Chao et al., 2020).

Leonurine is present in Leonurus japonicus Houtt. (Lamiaceae; Leonuri herba). Leonurine has been shown to attenuate Ang II-induced cardiomyocyte hypertrophy, fibrosis, and inflammation, and to preserve cardiac function (Shen et al., 2023) In the clinic, the intervention of 36 subjects using different doses of Leonurine revealed an increase in the relative abundance of Ruminococcus, Streptococcaceae, etc., and a relative abundance of Myobacterium, Veillonella, Lachnospiraceae, and Weissella was downward (Liao et al., 2021). Accumulating evidence suggests that high homocysteine and low methionine levels are associated with AS and myocardial infarction (Verhoef et al., 1996; Homocysteine Studies Collaboration, 2002; McCully, 2015). In the Liao et al. (2021) study, metabolomic analysis showed that Leonurine can affect homocysteine-methionine metabolism, increasing methionine levels.

Potential mechanisms of metabolites of Chinese botanical drugs affecting the intestinal mucosal barrier for the treatment of CVD are shown in Table 1.

**TABLE 1:** Lists of metabolites of Chinese botanical drugs acting on intestinal barrier damage in the treatment of CVD.

Plant metabolites	Source	Optional dose	Animal/cells	Targets on cardiovascular system	Targets on intestinal barrier	References
Resveratrol	Smilax glabra Roxb. [Smilacaceae; Smilacis glabrae rhizoma],	high fed diet with RSV (0.4%)	C57BL/6J mice; ApoE−/− mice	It inhibited FXR/FGF15 axis and increased CYP7A1, exerting an anti-atherosclerotic effect.	It decreased TMAO levels and increase the abundance of BSH-active bacteria such as *Lactobacillus* and Bifidobacterium.	Chen et al. (2016)
	Veratrum album L. [Melanthiaceae; Veratrum album]	50 mg/L	NG-nitro-L-arginine-methyl ester (L-NAME) and high-fat treatment-induced programmed hypertension	It attenuated L-NAME treatment-induced programmed hypertension.	It increased the abundance of the abundances of phylum Verrucomicrobia and genus Akkermansia Muciniphila.	Chen et al. (2019)
				It prevented the oxidative stress and actived AMP-activated protein kinase (AMPK)/peroxisome proliferator-activated receptor γ co-activator 1α (PGC-1α) pathway.		
Quercetin	Glehnia littoralis (A.Gray) F.Schmidt ex Miq. [Apiaceae; glehniae radix], Eucommia ulmoides Oliv. [Eucommiaceae; Eucommiae cortex], Panax notoginseng (Burkill) F.H.Chen [Araliaceae; notoginseng radix et rhizoma]	100 μg/day	HFD-fed mice	It attenuated weight gain and mitigated the extent of atherosclerotic lesions in the aortic sinus.	It decreased intestinal cholesterol, lysophosphatidic acids, and atherogenic lysophosphatidylcholine (LPC 18:1) levels while promoting an increased level of coprostanol.	Nie et al. (2019)
				It reduced malondialdehyde levels and elevated IL-6 levels to against immune/inflammatory and oxidative stress.	It enhanced Actinobacteria and Bacteroidetes populations, whereas markedly reduced Firmicutes abundance	
Baicalin	Scutellaria baicalensis Georgi [Lamiaceae; Scutellariae radix]	100 mg/kg	SHRs	It resulted in a partial decrease in blood pressure and reduce in serum hs-CRP, IL-1β, and IL-6	It decreased the ileal expression of Tlr2, HMGB1, RAGE, IL-1β, and IL-23.	Wu et al. (2019a)
					It mitigated intestinal hyperpermeability and systemic inflammatory response.	
					It increased the abundance of SCFA-producing fecal flora such as *Streptococcus*, Akkermansia, Allobaculum, Bifidobacterium, Lachnospiraceae_NK4B4_group, and Roseburia, as well as increased levels of SCFA.	
Naringin	Pericarpium Citri Reticulatae [Rutaceae; Citrus reticulata blanco], Paeonia lactiflora Pall. [Paeoniaceae; Paeoniae radix rubra]	100 mg/kg	ApoE−/− female mice	It enhanced bile acid synthesis through the FXR/FGF15-CYP7A1 pathway, resulting in reduced cholesterol levels and alleviation of atherosclerosis	It could promote the growth of 7α-dehydroxylase-producing bacteria and Eubacterium brachy and inhibits bile salt hydrolase-producing bacteria.	Wang et al. (2021b)
		50 μM	RIMVECs injury induced by TNF-α		It increased ZO-1, occludin, and claudin-1 of GVB.	Liu et al. (2020)
Naringenin	Pericarpium Citri Reticulatae [Rutaceae; Citrus reticulata blanco], Paeonia lactiflora Pall. [Paeoniaceae; Paeoniae radix rubra]	100 μM	RIMVECs injury induced by TNF-α		It restored occludin and claudin-1 expression levels.	Zhong et al. (2021)
					It inhibited NF-κB-mediated activation of the MLCK/p-MLC and TLR4/NF-κB/NLRxP3 pathways to repair the GVB.	
		100 μM	SMCs		It hyperpolarized SMCs through selective activation of BKCa channels, thereby reducing Ca2+ influx via VDCC and promoting SMCs relaxation for treating dyskinesia in rat colon.	Yang et al. (2014)
		1–100 μm	rat tail artery myocytes; endothelium-denuded rat aortic rings	It activates BKCa channel currents in rat tail artery myocytes.		Saponara et al. (2006)
				It had a relaxing effect on endothelium-denuded rat aortic rings that were precontracted with 20 mM KCl or norepinephrine.		
Notoginsenoside R1	Panax notoginseng (Burkill) F.H.Chen [Araliaceae; Notoginseng radix et rhizoma], Panax ginseng C.A.Mey. [Araliaceae; Ginseng radix et rhizoma]	25 mg/kg; 25 μM	MI/R mice model; CMs injury induced by H/R	It alleviates MI/R injury by nhibiting the TAK1/JNK/p38 signaling and attenuating cardiomyocyte apoptosis.		Zeng et al. (2023)
		100 μg/mL; 1 μg/g	lps-induced HUVECs and THP-1 cells injury; LPS-induced septic shock mice model	It inhibited the LPS-induced I kappa B-alpha and TNF-alpha and prevented the upregulation of PAI-1 antigen, exerting anti-inflammatory and antithrombotic effects.		Zhang et al. (1997)
		10 mg/kg	intestinal I/R rat model		It attenuated intestinal I/R-induced microvascular hyperpermeability.	Li et al. (2014)
					It reduced inflammatory cytokine, activated the NF-κB pathway and increased tight junction proteins, and improved energy metabolism in jejunum of.	
Ginsenoside Rc	Panax ginseng C.A.Mey. [Araliaceae; Ginseng radix et rhizoma]	40 mg/kg	HFD-induced ApoE−/− mice	It decreased TC, TG, and LDL-C levels and increased HDL-C levels, and reduced inflammatory cytokines TNF-α, IL-6, and IL-1β.	It reduced the relative abundance of Faecalibaculum, Oscillibacter, Eubacterium_coprostanoligenes_group, and Blautia, while increased the relative abundance of Muribaculaceae, *Lactobacillus*, Ileibacterium, and Bifidobacterium.	Xie et al. (2022)
					It changed the purine metabolism pathway, the TCA cycle, and the primary bile acid biosynthesis pathway.	
Berberine	Coptis chinensis Franch. [Ranunculaceae; Coptidis rhizoma], Phellodendron amurense Rupr. [Rutaceae; phellodendri chinensis cortex], Uncaria rhynchophylla (Miq.) Miq. [Rubiaceae; Uncariae ramulus cum uncis]	40 mg/kg	deoxynivalenol -Induced Intestinal barrier dysfunction		It increased the expression of serum antioxidant enzymes and T-cell surface antigens and by decreasing the release of pro-inflammatory cytokines in the small intestine.	Tang et al. (2021)
					It increased ileal and jejunal mucosal ZO-1 by decreasing the expression of ERK, JNK, and NF-κB, occludin and claudin-1 protein expression levels in the jejunum and ileum, and improved jejunal morphology.	
		50 mg/kg	HFD-fed ApoE−/− mice	It reduced AS area and lipid levels, decreased pro-inflammatory cytokines TNF-α, IL-1β, IL-6, and increased anti-inflammatory IL-10 and lipocalin levels.	It altered the abundance of Roseburia, Blautia, Allobaculum, Alistipes, Turicibacter, and Bilophila.	Wu et al. (2020)
		0.5 g bid	AS patients	Patients fecal and stool levels of both TMA and TMAO were significantly reduced along with a notable decrease in plaque score.	It decreased Eubacterium_hallii_group, Anaerostipes, Faecalibacterium, Dialister, Eubacterium_coprostanoligenes_group, Coprococcus_3, Butyricicoccus and *Clostridium*_sensu_strito_1.	Ma et, al., 2022
		25, 50, and 75 mg/kg	NSAID-Induced Intestinal barrier dysfunction		It upregulated the expression of PGP9.5, GFAP, and GDNF with repair of the enteric nervous system.	Chao et al. (2020)
Leonurine	Leonurus japonicus Houtt. [Lamiaceae; Leonuri herba]	10 mg/kg, and 20 mg/kg	ang II-induced cardiac remodeling in mice and cardiomyocyte cell line H9c2	It alleviated cardiac hypertrophy, fibrosis, and inflammation in both mice and cultured cardiomyocytes. Echocardiography revealed that leonurine preserved cardiac function in mice.		Shen et al. (2023)
		50mg, 150mg, and 300 mg	participants in the leonurine phase I clinical trial	It affected homocysteine-methionine metabolism and increases methionine levels.	It increased the abundance of Ruminococcus, Streptococcaceae and decreased the abundance of Myobacterium, Veillonella, Lachnospiraceae, and Weissella.	Liao et al. (2021)

## Potential mechanisms of Chinese medicine formulas affecting intestinal mucosal barrier for the treatment of CVD

Shen-Fu Decoction (SFD), a classic traditional Chinese formula, is composed of Panax ginseng C.A.Mey. (Araliaceae; Ginseng radix et rhizoma), Aconitum carmichaelii Debeaux (Ranunculaceae; Aconiti lateralis radix praeparata). SFD significantly reduced the mortality rate of sepsis model induced by cecal ligation and puncture (CLP), prevented intestinal and liver injury, alleviated increased intestinal permeability and inflammation, and impaired intestinal barrier by regulating the expression of ZO-1, occludin, claudin-1, and Phosphorylated Vasodilator Stimulated Phosphoprotein (p-VASP) (Liu et al., 2021b). Yan et al. (2018) demonstrated that the regulation of Fas, Fas-L, Bcl-2, and Bax proteins in the apoptotic pathway by SFD and microRNAs played a pivotal role in ameliorating cardiac function and hemodynamic indices in rats with HF. Interestingly, another formula containing Ren Shen and Fu Zi, Fuzi decoction (FZD), has also been shown to have therapeutic effects on CHF. Gao et al. (Gao et al., 2022) found that FZD improved cardiac function and reduced the effects of pyruvate and lactate. Recent studies have shown that the pyruvate-lactate axis is critical for cardiac homeostasis (Cluntun et al., 2021) Gao et al. (2023) also found that FZD increased the Firmicutes-Bacteroidetes ratio and the abundance of *Lactobacillus*, and affected the β-diversity of the intestinal microbiota in rats with CHF. FZD increased the Firmicutes-Bacteroidetes ratio and *Lactobacillus* abundance and affected the β-diversity of the gut microbiota in rats with CHF (Gao et al., 2023). Non-targeted metabolomics analysis showed that FZD affected the biosynthesis of valine, leucine, and isoleucine. It increased the levels of acetic acid, propionic acid, butyric acid, and isovaleric acid in the feces of CHF rats. FMT demonstrates once again that the mechanism of action of FZD in the treatment of CHF may be related to the improvement of the gut microbiota, the elevation of SCFA content, and the realization of the amino acid pathway (Gao et al., 2023).

Lingguizhugan decoction (LGZGD), an ancient TCM formula from the Treatise on Cold Pathogenic and Miscellaneous Diseases, was prepared from Poria cocos (Schw.) Wolf (Polyporaceae; Poria), Neolitsea cassia (L.) Kosterm. (Lauraceae; Cinnamomi ramulus), Atractylodes macrocephala Koidz. (Asteraceae; Atractylodis macrocephalae rhizoma), Glycyrrhiza uralensis Fisch. ex DC. (Fabaceae; Glycyrrhizae radix et rhizoma). In DOX-induced myocardial injury in rats, LGZGD was found to ameliorate cardiac dysfunction and attenuate myocardial morphological injury and mitochondrial damage. LGZGD downregulated miR-24 expression, upregulated junctophilin-2 (JP-2) expression, and antagonized T-tubule-sarcoplasmic reticulum (TT-SR) microstructural remodeling, thereby improving cyclic Ca2+ transients and cell contraction (Li et al., 2019). In transverse aortic constriction (TAC)-induced HF mice, LGZGD significantly ameliorated cardiac dysfunction by downregulating p38 and ERK and bi-directionally regulating the expression of cardiac hypertrophy-related genes and proteins such as protein kinase B (AKT)- Glycosynthase kinase 3β (GSK3β)/mammalian target of rapamycin (mTOR)/P70S6K in TAC mice (Chen et al., 2022).

Buyang Huanwu Decoction (BYHWD) is composed of Astragalus mongholicus Bunge [Fabaceae; Astragali radix], Angelica sinensis (Oliv.) Diels [Apiaceae; Angelicae sinensis radix], Radix Paeonia lactiflora Pall. [Paeoniaceae; Paeoniae radix rubra], Pheretima Aspergillum (E.Perrier) [Megascolecidae; Pheretima], Rhizoma Ligustici Chuanxiong [Apiaceae; Chuanxiong rhizoma], Carthamus tinctorius L. [Asteraceae; Carthami Flos], Prunus persica (L.) Batsch [Rosaceae; Persicae semen] (Chen et al., 2020). Clinical studies have shown that BYHWD reduced levels of hs-CRP, TNF-α, IL-18, and TMAO, increased the abundance of *Lactobacillus* and Bifidobacterium, and reduced the abundance of *Escherichia coli* and fungi in patients with stable coronary artery disease (Liang et al., 2023). *In vitro* assays showed that BYHWD reduced the expression of IL-6, IL-1β, and matrix metalloproteinase 9 (MMP9), increased cell migration, decreased the proportion of RCFs in the S + G2 phase, and inhibited cell division and proliferation, thereby inhibiting myocardial fibrosis (Wang et al., 2022). The animal experiments demonstrated that BYHWD improved myocardial and cardiac function in HF rats, while also reducing serum d-lactate and TMAO levels. Furthermore, it increased the expression of occludin and claudin-1. Additionally, the taxonomic composition of gut microbiota was altered, with an observed increase in *Lactobacillus* abundance and a concurrent decrease in Romboutsia abundance following treatment with BYHWD in HF rats. These findings suggest that BYHWD has potential therapeutic implications for heart failure through its ability to restore intestinal mucosal barrier integrity (Weng et al., 2023).

Taohong Siwu Decoction (THSWD) consists of Rehmannia glutinosa (Gaertn.) DC. [Orobanchaceae, Rehmanniae radix praeparata], Angelica sinensis (Oliv.) Diels [Apiaceae; Angelicae sinensis radix], Radix Paeonia lactiflora Pall. [Paeoniaceae; Paeoniae radix rubra], Rhizoma Ligustici Chuanxiong [Apiaceae; Chuanxiong rhizoma], Carthamus tinctorius L. [Asteraceae; Carthami Flos], Prunus persica (L.) Batsch [Rosaceae; Persicae semen]. It is widely used in blood stasis type CVD (Liu et, al., 2022a; Luo et, al., 2019; Xia et, al., 2021). As found in a rat model of MI, THSWD administration improved cardiac function in MI rats. This study suggests that THSWD significantly increased the expression of basic bFGF, insulin-like growth factor-1 (IGF-1), reduced collagen deposition, promoted angiogenesis, reduced apoptosis, and activated the phosphatidylinositol 3-kinase (PI3K)/AKT signaling pathway. Notably, THSWD significantly reduced mitochondrial ROS production and inhibited excessive mitochondrial fission. Beneficial changes in the fecal microbiota of rats also occurred after THSWD intervention, as evidenced by increased Firmicutes/Bacteroidetes (F/B) value and increased *Lactobacillus*.

Potential mechanisms of Chinese medicine formulas affecting intestinal mucosal barrier for the treatment of CVD are shown in Table 2.

**TABLE 2:** Lists of Chinese medicine formulas acting on intestinal barrier damage in the treatment of CVD.

Chinese medicine formula	Composition	Extraction	Optimal dose	Model	Targets on cardiovascular system	Targets on intestinal barrier	References
Shen-Fu decoction	Panax ginseng C.A.Mey. [Araliaceae; Ginseng radix et rhizoma], Aconitum carmichaelii Debeaux [Ranunculaceae; Aconiti lateralis radix praeparata]	Mix in a ratio of 30: 15 g in sequence.	6 mg/kg	sepsis model induced by CLP		It alleviated LPS, FITC-dextran, D-lactate, TNF-α and IL-6 by regulating the expression of ZO-1, occludin, claudin-1, and p-VASP.	Liu et al. (2021a)
		SFD formulation, with a total weight of 45 g, were dissolved in 45 mL distilled water to a final concentration of 1 g/mL for experimental use.					
		Mix in a ratio of 10: 10 g in sequence.	2.67 g/kg	heart failure rat model left induced by coronary artery ligation	It could regulate the proteins Fas, Fas-L, Bcl-2, and Bax as well as microRNAs in the apoptotic pathway to enhance cardiac function.		Yan et al. (2018)
		Extraction process wasn’t provided.					
Fuzi decoction	Aconitum carmichaelii Debeaux [Ranunculaceae; Aconiti lateralis radix praeparata], Panax ginseng C.A.Mey. [Araliaceae; Ginseng radix et rhizoma], Atractylodes macrocephala Koidz. [Asteraceae; Atractylodis macrocephalae rhizoma], Poria cocos (Schw.) Wolf [Polyporaceae; Poria], Paeonia lactiflora Pall. [Paeoniaceae; Paeoniae radix alba]	Mix in a ratio of 15: 6: 12: 9: 9 g in sequence.	5.1 and 20.4 g/kg	heart failure rat model induced by abdominal aortic coarctation	It improved cardiac function and reduced the effects of pyruvate and lactate.	It increased the Firmicutes-Bacteroidetes ratio and *Lactobacillus* abundance and affected the β-diversity of the intestinal microbiota in rats with CHF.	Gao et al. (2022); Gao et, al. (2023)
		The mixture was extracted twice by heating and reflux with 10 volumes of 70% ethanol for 2 h eachtime. The drug solution was filtered and evaporated to near dryness in a water bath at 60°C with rotation.				It affected the biosynthesis of valine, leucine, and isoleucine. And it increased the levels of acetic acid, propionic acid, butyric acid and isopentanoic acid in the feces of CHF rats.	
Buyang Huanwu Decoction	Astragalus mongholicus Bunge [Fabaceae; Astragali radix], Angelica sinensis (Oliv.) Diels [Apiaceae; Angelicae sinensis radix], Radix Paeonia lactiflora Pall. [Paeoniaceae; Paeoniae radix rubra], Pheretima Aspergillum (E.Perrier) [Megascolecidae; Pheretima], Rhizoma Ligustici Chuanxiong [Apiaceae; Chuanxiong rhizoma], Carthamus tinctorius L. [Asteraceae; Carthami Flos], Prunus persica (L.) Batsch [Rosaceae; Persicae semen]	Mix in a ratio of 20: 15: 15: 15: 12: 10: 10 g in sequence.	100 mL bid	patients with stable coronary artery disease	It reduced levels of hs-CRP, TNF-α, IL-18, and TMAO.	It increased the abundance of *Lactobacillus* and Bifidobacterium, and reduced the abundance of *Escherichia coli* and fungi.	Liang et al. (2023)
		All botanical drugs were soaked in cold water for 30 min, decoct twice, extract the juice and mix well, concentrate to 200 mL.					
		Mix in a ratio of 60: 10: 10: 10: 10: 5: 5 g in sequence.	10% pharmaceutical serum extracted from rats fed with 1 mL/kg BYHWD	cardiac fibroblasts rat model	It reduced the expression of IL-6, IL-1β, and matrix MMP9, inhibited cell division and proliferation to inhibit myocardial fibrosis.		Wang et al. (2022)
		All botanical drugs were soaked for 2 h, boiled for 0.5 h over a military fire, slowly decocted for 1 h on a civilian fire, and then filtered. The above steps were repeated once. All filtrate was combined and concentrated using a rotary evaporator to achieve a final raw drug mass concentration of 2 g/mL					
		Mix in a ratio of 120: 6: 4.5: 3: 3: 3: 3 g in sequence.	15.02 g/kg	left anterior descending coronary artery ligation induced HF rat model	It improved heart function and relieved histological injury in the myocardium. of rats with HF.	It reduced serum d-lactate and TMAO levels and increased occludin and claudin-1 expression to repair the intestinal mucosal barrier.	Weng et al. (2023)
		All botanical drugs were soaked in deionized water for 30 min, then decocted twice in distilled water (10 times the weight of the botanical drugs) for 30 min and filtered. The extraction solution was further mixed and concentrated to obtain the BYHWD solution at a concentration of 2.14 g/mL (raw botanical drugs).				It increased *Lactobacillus* and reduced Romboutsia.	
Taohong Siwu Decoction	Rehmannia glutinosa (Gaertn.) DC. [Orobanchaceae, Rehmanniae radix praeparata], Angelica sinensis (Oliv.) Diels [Apiaceae; Angelicae sinensis radix], Radix Paeonia lactiflora Pall. [Paeoniaceae; Paeoniae radix rubra], Rhizoma Ligustici Chuanxiong [Apiaceae; Chuanxiong rhizoma], Carthamus tinctorius L. [Asteraceae; Carthami Flos], Prunus persica (L.) Batsch [Rosaceae; Persicae semen]	Mix in a ratio of 11.19: 14.92: 5.6: 3.73: 3.73: 3.78 g in sequence.	5, 10, 20 g/kg	rat model with blood deficiency and blood stasis syndrome	It increased the expression of bFGF, IGF-1, reduced collagen deposition, promoted angiogenesis, reduced apoptosis, and activated PI3K/AKT signaling pathway.	It increased F/B value and the abundance of *Lactobacillus*.	He et al., 2023
		The medicinal materials were soaked in 8 times the amount of water for 30 min, decocted for 1 h for the first time, decocted for 1.5 h for the second time, combined the two decoctions, concentrated under reduced pressure, the concentrated liquid was fixed to 43 mL (1 g crude drug amount/mL) to obtain THSWD.			It reduced mitochondrial ROS production and inhibited excessive mitochondrial fission.		

## Potential mechanisms of Chinese patent medicine affecting intestinal mucosal barrier for the treatment of CVD

CPM has been extensively utilized in the secondary prevention and treatment of CVD (Chen et al., 2023a; Shang et al., 2009). Tongxinluo (TXL), is an approved CPM by the State Food and Drug Administration of China for angina pectoris and ischemic stroke (Qi et al., 2022). Qi et al. (2022) found TXL could enhance atherosclerotic plaque stability, suppress NLRP3, caspase-1, IL-1β, and IL-18 inflammatory pathways expression as well as increase the relative abundance of F/B ratio, Alipipes, *Campylobacter*, Rikenella, indistinctus, viscericola, nordii, subantarcticus, and other bacteria. The fecal metabolites erucic acid, N-acetylneuraminate, chenodeoxycholate, and 6-tuliposide B in the TXL intervention group were significantly reduced, while hydroxyphenyllactic acid, trans-ferulic acid (TFA), and others were significantly increased. This study found that some metabolites were significantly associated with some bacteria and some inflammatory factors. In particular, the metabolite trans-ferulic acid transcarboxylate inhibits the NLRP3 inflammatory pathway (Qi et al., 2022). Trans-ferulic acid, the major active form of ferulic acid (Cai et al., 2006), has been shown to reduce oxidative stress, apoptosis, and necrosis in peripheral monocytes (Perez-Ternero et al., 2017), and is involved in the activation of classical inflammatory pathways such as PPARγ and NF-κB//NLRP3 (Mahmoud et al., 2019). This evidence suggests that TXL may achieve improved plaque stability and inflammation in rabbits with atherosclerotic AS by regulating gut microbiota and metabolism (Qi et al., 2022). Another study demonstrated that TXL treatment attenuated intestinal I/R-induced mast cell activation, restrained overexpression of TLR4, NF-κB, TNF-α, platelet endothelial cell adhesion molecular (PECAM), High Mobility Group Protein 1 (HMGB1) expression, and protected VE-cadherin and Angiopoietin Like protein 4 (ANGPTL4) proteins to repair microvascular dysfunctionand endothelial injury (Zhang et al., 2020a; Zhang et al., 2018).

Qiliqiangxin (QL) capsules contain Astragalus mongholicus Bunge [Fabaceae, Astragali radix], Panax ginseng C.A.Mey. [Araliaceae; Ginseng radix et rhizoma], Aconitum carmichaelii Debeaux [Ranunculaceae; Aconiti lateralis radix praeparata], Salvia miltiorrhiza Bunge [Lamiaceae; Salvia miltiorrhizae radix et rhizoma], Descurainia sophia (L.) Webb ex Prantl [Brassicaceae, Descurainiae semen lepidii semen], etc. QL, also a CPM for the treatment of HF, reduced protein expression levels of myocardial NF-κB, NLRP3, ASC, caspase-1, and cleaved-IL-1β, preserved cardiac function, and significantly improved LV remodeling. In addition, QL treatment of HF rats resulted in increased *Lactobacillus*, improved intestinal villus edema, breakage or absence, and gaps between epithelial cells (Lu et al., 2022). In addition, Qiliqiangxin therapeutically protects the heart from ventricular remodeling and HF by modulating the microbiota of Paraprevotella, Phascolarctobacterium, and Intestinimonas and repairing the intestinal mucosal barrier (Lu et al., 2022).

Xuesaitong (XST) primarily consists of Panax notoginseng saponin (PNS), which exhibits dual effects of hemostasis and angiogenesis, making it extensively utilized for the treatment of cardiovascular and cerebrovascular diseases (Liao et al., 2023). Angiogenesis plays a crucial role in the reparative process following MI injury (Wu et al., 2021). In a mice model of MI, XST was demonstrated to enhance myocardial angiogenesis and mitigate myocardial fibrosis by specifically targeting nuclear hormone receptor77(Nur77) to suppress miR-3158-3p (Liao et al., 2023). Additionally, XST exhibited the ability to upregulate the expression of the VEGF signaling pathway, thereby promoting angiogenesis (Wang et al., 2012). Proteomic and cellular investigations unveiled that XST-induced alterations in protein levels of myocardial pyruvate dehydrogenase E1 alpha (PDHA1), hydroxyacyl-coenzyme A dehydrogenase (HADHA), peroxiredoxin 3 (PRX3), gamma-enolase, acetyl-coenzyme A acyltransferase 2 (ACAA2). In addition, XST promoted the activity of PDH, a crucial enzyme associated with the tricarboxylic acid (TCA) cycle, and enhanced intracellular levels of acetyl coenzyme A and ATP effectively mitigated malondialdehyde (MDA) release in cardiomyocytes (Zhao et al., 2017). Xu et al. investigated the impact of XST on intestinal barrier dysfunction and its underlying mechanisms in a rat model of intestinal I/R injury. XST demonstrated significant efficacy in alleviating I/R-induced intestinal barrier dysfunction by suppressing TNF-α expression, upregulating Bcl-2, downregulating caspase-3, and promoting intestinal peristalsis (Xu et al., 2015).

Zheng et al. (2023) conducted a randomized controlled trial in patients with acute ST-segment elevation myocardial infarction after percutaneous coronary intervention (PCI) and found that Tongguan capsules were able to improve the ventricular remodeling of patients. In addition, Tongguan capsules adjust the abundance of relevant genera of intestinal Prevotella, Agaricus, Microbacterium, and Enterococci, which increases the beneficial bacterial colonization and bacterial diversity, as well as adjust the structure of gut microbiota.

Shenfu injection, comprising ginsenosides and aconite alkaloid, exhibited enhanced hemodynamics and sustained intragastric pH in a myocardial I/R model, thereby preventing inadequate perfusion of the gastrointestinal microcirculation following myocardial I/R (Zhang et al., 2006). In children undergoing heart surgery for congenital heart defects, Shenfu injection significantly reduced the decrease in gastric mucosal pH during surgery. It also decreased plasma levels of diamine oxidase (DAO), MDA, LPS, IL-6, and creatine kinase isoenzyme MB. Additionally, Shenfu injection reduced postoperative utilization of positive inotropic drugs and length of stay in the care unit. Therefore, Shenfu injection attenuated gastrointestinal tract injury and suppressed inflammatory response after extracorporeal circulation in patients with congenital heart disease (Xia et al., 2005).

Potential mechanisms of Chinese patent medicine affecting intestinal mucosal barrier for the treatment of CVD are shown in Table 3.

**TABLE 3:** Lists of Chinese patent medicine acting on intestinal barrier damage in the treatment of CVD.

Chinese patent medicine	Composition	Optimal dose	Model	Targets on cardiovascular system	Targets on intestinal barrier	References
Tongxinluo	Panax ginseng C.A.Mey. [Araliaceae; Ginseng radix et rhizoma], Hirudo nipponica Whitman [Hirudinidae; Hirudo], Scolopendra subspinipes mutilans L. Koch [Scolopendridae; Scolopendra], Eupolyphaga sinensis Walker [Corydidae; Eupolyphaga steleophaga], Buthus martensii Karsch [Buthidae; Scorpio], Cryptotympana pustulata Fabricius [Cicadidae; Cicadae periostracum], Paeonia lactiflora Pall. [Paeoniaceae; Paeoniae radix rubra], Camphora officinarum Nees [Lauraceae; Borneolum], Santalum album L [Santalaceae; Santali albi lignum], Boswellia carterii Birdw. [Burseraceae, Olibanum], Dalbergia odorifera T.C.Chen [Fabaceae; Dalberglae odoriferae lignum], Ziziphus jujuba Mill [Rhamnaceae, Ziziphi spinosae semen]	0.6 g/kg	rabbit with balloon-induced aortic endothelium injury and HFD-fed	It enhanced atherosclerotic plaque stability, suppress NLRP3, caspase-1, IL-1β, and IL-18 inflammatory pathways expression.	It increased the relative abundance of Firmicutes/Bacteroidetes (F/B) ratio, Alipipes, *Campylobacter*, Rikenella, indistinctus, viscericola, nordi, subantarcticus, and other bacteria.	Qi et al. (2022)
					It increased metabolites such as trans-ferulic acid, which inhibited NLRP3 inflammatory pathways expression	
		0.4, 0.8, and 1.6 g/kg	obstructing superior mesenteric artery induced intestinal I/R rat model		It attenuated intestinal I/R-induced mast cell activation, restrained overexpression of TLR4, NF-κB, TNF-α, PECAM-1, HMGB1 expression, and protected VE-cadherinand ANGPTL4 proteins to repair microvascular dysfunctionand endothelial injury.	Zhang et al. (2018);
						Zhang et al. (2020b)
Qiliqiangxin	Astragalus mongholicus Bunge [Fabaceae, Astragali radix], Panax ginseng C.A.Mey. [Araliaceae; Ginseng radix et rhizoma], Aconitum carmichaelii Debeaux [Ranunculaceae; Aconiti lateralis radix praeparata], Salvia miltiorrhiza Bunge [Lamiaceae; Salvia miltiorrhizae radix et rhizoma], Descurainia sophia (L.) Webb ex Prantl [Brassicaceae, Descurainiae semen lepidii semen], Alisma plantago-aquatica subsp. orientale (Sam.) Sam. [Alismataceae; Alismatis rhizoma], Neolitsea cassia (L.) Kosterm. [Lauraceae; Cinnamomi ramulus], Periplocae cortex, Periploca sepium Bunge [Apocynaceae; Periplocae cortex], Polygonatum sibiricum Redouté [Asparagaceae; Polygonati rhizoma], Carthamus tinctorius L. [Asteraceae; Carthami Flos], Polygonatum odoratum (Mill.) Druce [Asparagaceae; Polygonati odorati rhizoma], Citrus reticulata Blanco [Rutaceae; Citri reticulatae pericarpium]	100 mg/kg	TAC induced HF rat model	It reduced protein expression levels of myocardial NF-κB, NLRP3, ASC, caspase-1, and cleaved-IL-1β, preserved cardiac function, and improved LV remodeling.	It resulted in increased *Lactobacillus*, improved intestinal villus edema, breakage or absence, and gaps between epithelial cells.	Lu et al. (2022)
					It protected the heart from ventricular remodeling and HF by modulating the flora of Paraprevotella, Phascolarctobacterium, and Intestinimonas and repairing the intestinal mucosal barrier.	Lu et al. (2022)
Xuesaitong	Panax notoginseng saponin	40 mg/kg	coronary artery ligation induced MI/R rat model	It enhanced myocardial angiogenesis and mitigated myocardial fibrosis by specifically targeting Nur77 to suppress miR-3158-3p.		Liao et al. (2023)
		400 mg/kg	coronary artery ligation induced MI rat model	It upregulated VEGF signaling pathway to promoting angiogenesis.		Wang et al. (2012)
		80 mg/kg;	coronary artery ligation induced MI/R rat model; H9c2 cells with H/R injury	It induced alterations in protein levels of myocardial PDHA1, HADHA, PRX3, gamma-enolase, ACAA2.		Zhao et al. (2017)
		100, 200, 400 μg/mL		It promoted the activity of PDH and enhancing intracellular levels of acetyl coenzyme A and ATP, effectively mitigated MDA release in cardiomyocytes.		
		6.58 mg/kg	obstructing superior mesenteric artery induced intestinal I/R rat model		It alleviated I/R-induced intestinal barrier dysfunction by decreasing TNF-αexpression, upregulating Bcl-2, downregulating caspase-3, and promoting intestinal peristalsis.	Xu et al. (2015)
Tongguan Capsules	Astragalus mongholicus Bunge [Fabaceae; Astragali radix], Salvia miltiorrhiza Bunge [Lamiaceae; Salvia miltiorrhizae radix et rhizoma], Gardenia jasminoides var. grandiflora [Hirudo, Paeoniae radix rubra], Camphora officinarum Nees [Lauraceae; Borneolum]	4.5 g tid	acute ST-segment elevation myocardial infarction after PCI	It improved the ventricular remodeling of patients.	It adjusted the abundance of relevant genera of intestinal Prevotella, Agaricus, Microbacterium, and Enterococci.	Zheng et al. (2023)
Shen-Fu injection	0.9 mg ginsenosides and 0.1 mg aconite alkaloid per milliliter	5, 10, 20 mL/kg	coronary artery ligation induced MI rat model	It enhanced hemodynamics.	It sustained intragastric pH in a myocardial I/R model to prevent inadequate perfusion of the gastrointestinal microcirculation.	Zhang et al. (2006)
		1.5 mL/kg	cardio-pulmonary bypass in patients undergoing cardiac surgery		It reduced the decrease in gastric mucosal pH during surgery.	Xia et al. (2005)
					It also decreased plasma levels of DAO, MDA, LPS, IL-6 and creatine kinase isoenzyme MB.	

## Potential mechanisms of TCM affecting intestine to treat CVD risk factors

Fasting hyperglycemia, hypertriglyceridemia, low HDL cholesterol, hypertension, and/or metabolic disorders such as abdominal obesity and nonalcoholic fatty liver disease are risk factors for the development of coronary heart disease (CHD) and cardiovascular events (Lakka et al., 2002). RV intake decreased the relative abundance of Turicibacteraceae, Moryella, Lachnospiraceae, and Akkermansia and increased the relative abundance of *Bacteroides* and Parabacteroides in obese mice. And fecal FMT of healthy resveratrol-fed mice improved glucose homeostasis in obese mice (Sung et al., 2016).RV may indirectly treat CVD by modulating the microbial structure and improving lipid and glucose metabolism.

Porras et al. (Porras et al., 2017) identified the ability of quercetin to regulate lipid metabolism genes such as LXRα, sterol regulatory element binding protein (SREBP)-1c, fatty acid synthase (FAS), fatty acid binding protein (FABP)1 and FAT/CD36 and other lipid metabolism genes expression to regulate cytochrome P450 2E1 (CYP2E1)-dependent lipid peroxidation and related lipotoxicity, thereby reducing intrahepatic lipid accumulation and hence insulin resistance. Quercetin restored gut microbiota imbalance, SCFAs in HFD-fed mice, occludin, claudin 1, and intestinal alkaline phosphatase expression (Porras et, al., 2017). In addition, the associated endotoxemia-mediated TLR-4 pathway was also inhibited by quercetin, which subsequently inhibited inflammasome response and reticular stress pathway activation and blocked lipid metabolism gene expression (Porras et al., 2017).

Lu et al. (2020) demonstrated that Neohesperidin (Neo) attenuated the levels of inflammatory factors, including TNF-α, monocyte chemoattractant protein-1 (MCP-1), and IL-1β while modulating the expression of lipid transporter genes, adipogenic genes, and fatty acid oxidation in epididymal white adipose tissue. Additionally, Neo improved intestinal barrier integrity and reduced serum metabolic LPS levels in obese mice. However, eWAT was shown to be an exosome-dependent promoter of Ang II-induced cardiac fibrosis and subsequent cardiac dysfunction (Su et al., 2022). The study by Lu et al. also found that Neo neo-administration induced significant alterations in the composition of gut microbiota in high-fat diet-fed mice, characterized by a decrease in Faecalibaculum and an increase in Blautia, Mucispirillum, Lachnospiraceae_ UCG-006, *Streptococcus*, Enterorhabdus, and *Bacteroides*. Furthermore, FMT with Neo-treated samples effectively ameliorated obesity and metabolic disorders observed in HFD-fed mice (Lu et al., 2020).

Ginsenoside Rb1 (Rb1) oral supplementation ameliorated dyslipidemia, improved insulin sensitivity in HFD-induced obese mice, and reversed the expression of uncoupling protein 2 (UCP2), Nuclear receptor subfamily one group H member 4 (Nr1h4), and Fiaf. Rb1 altered gut microbiota composition and increased Akkermansia spp. abundance, and maintains amino acid metabolic homeostasis, particularly leucine (Leu), isoleucine (Iso), tryptophan (Trp), and alanine (Ala). Correlation analyses revealed that Akkermansia spp. alteration of the Ala gut microbiota and regulation of amino acid metabolism may be the mechanism by which Rb1 acts (Yang et al., 2021).

BYHWD was able to inhibit body fat accumulation and blood triglyceride levels in HFD rats significantly increase Bacteroidetes and dramatically decrease Firmicutes at the phyla level, and the remarkable increase in the abundance of *Lactobacillus* and Blautia (Liu et al., 2022b). Glycoside metabolites (astragaloside IV, paeoniflorin, and amygdalin) in BYHWD were shown to alleviate atherosclerotic inflammation. These glycosides were able to reduce pro-inflammatory factors and adhesion molecules, decrease the number of foam cells and intracellular lipid content, and prevent macrophage inflammation, all by inhibiting the activation of the Janus Kinase (JAK)/signal transducer and activator of transcription (STAT) pathway (Fu et al., 2022).

Lingguizhugan decoction (LGZGD) is an ancient Chinese herbal formula from the Treatise on Cold Pathogenic and Miscellaneous Diseases, consisting of Poria cocos (Fu Ling), Ramulus Cinnamomi (Gui Zhi), Atractylodis Macrocephalae Rhizoma (Bai Zhu), and Radix Glycyrrhizae (Gan Cao). It was found in the HFD rat model that LGZGD treatment not only reduced plasma insulin concentration, glucose tolerance, and lipids but also restored small intestinal villus morphology and upregulated occludin levels. This may be achieved by upregulating the relative abundance of Akkermansia, Faecalibacterium, and Phascolarctobacterium (Ning et al., 2022).

Potential mechanisms of TCM affecting intestine to treat CVD risk factors are shown in Table 4.

**TABLE 4:** Lists of TCM acting on intestinal barrier damage in the treatment of CVD risk factors.

metabolites of	Source	Optional dose	Animal/cells	Targets on cardiovascular system	Targets on intestinal barrier	References
Resveratrol	Smilax glabra Roxb. [Smilacaceae; Smilacis glabrae rhizoma], Veratrum album L. [Melanthiaceae; Veratrum album]	high-fat/high-sugar diet with RSV (0.4%)	HFHS-fed mice	It improved glucose clearance.	It increased the abundance of *Bacteroides* and Parabacteroides, and decreased the abundance of Turicibacteraceae, Moryella, Lachnospiraceae and Akkermansia.	Wang et al. (2018)
Quercetin	Glehnia littoralis (A.Gray) F.Schmidt ex Miq. [Apiaceae; glehniae radix], Eucommia ulmoides Oliv. [Eucommiaceae; Eucommiae cortex], Panax notoginseng (Burkill) F.H.Chen [Araliaceae; notoginseng radix et rhizoma]	fed diet with 0.05% (wt/wt) aglycone quercetin	HFD-fed mice	It regulated LXRα, SREBP-1c, FAS, FABP1 and FAT/CD36, and lipid metabolism gene expression, CYP2E1-dependent lipid peroxidation and related lipotoxicity to reduce intrahepatic lipid accumulation.	It reduced Desulfovibrio and *Helicobacter*, and restored SCFAs, occludin, claudin 1, and intestinal alkaline phosphatase expression.	Porras et al. (2017)
				It inhibited TLR-4 pathway to inhibit inflammasome response and reticular stress pathway activation and blocked lipid metabolism gene.		
Neohesperidin	Citrus aurantium F. aurantium [Rutaceae; Fructus aurantii], Citrus reticulata Blanco [Rutaceae; Citrus reticulata pericarpium], Magnolia Officinalis Rehd Et Wils. [Magnoliaceae; Mangnolia officinalis cortex]	50 mg/kg	HFD-fed mice	It decreased TNF-α, MCP-1, and IL-1β.	It increased ZO-1 and Occludin, and reduced IL-1β and TNF-α in colon.	Lu et al. (2020)
				It increased SREBP-1c and FAS in the.	It reduced the levels of serum endotoxin.	
					It decreased Faecalibaculum and increase Blautia, Mucispirillum, Lachnospiraceae_ UCG-006, *Streptococcus*, Enterorhabdus, and *Bacteroides*.	
Ginsenoside Rb1	Panax ginseng C.A.Mey. [Araliaceae; Ginseng radix et rhizoma]	200 mg/kg	HFD-fed mice	It ameliorated dyslipidemia, improved insulin sensitivity, and reversed the expression of UCP2, Nr1H4, and Fiaf.	It increased Akkermansia spp. abundance and maintains amino acid metabolic homeostasis, particularly Leu, isoleucine, Iso, Trp, and Ala.	Yang et al. (2021)

Chinese medicine formula	Composition	Extraction	Optimal dose	Model	Targets on cardiovascular system	Targets on intestinal barrier	References
Buyang Huanwu Decoction	same as TABEL 2	Mix in a ratio of 120: 6: 4.5: 3: 3: 3: 3 g in sequence.	7.83 g/kg	obese T2DM model	It inhibited body fat accumulation and blood triglyceride levels in HFD rats.	It increased Bacteroidetes and dramatically decreased Firmicutes at the phyla level and increased the abundance of *Lactobacillus* and Blautia at the genus level.	Liu et al. (2022a)
		10 doses botanical drugs were mixed and soaked in distilled water for 1 h and then boiled twice. The boiling time was set to 1.5 h and 1 h respectively. After two boils or extractions, the water mixture was filtered and freeze-dried.					
		Mix in a ratio of 60: 9: 9: 9: 6: 9: g in sequence.	BYHWD 2.772 g/kg; glycosides of BYHWD 0.167 g/kg and 0.084 g/kg	HFD-fed mice	It reduced pro-inflammatory factors and adhesion molecules, decrease the number of foam cells and intracellular lipid content, and prevent macrophage inflammation, all by inhibiting the activation of the JAK/STAT pathway		Fu et al. (2022)
		All botanical drugs were immersed and extracted two times. The two extracts were mixed concentrated and dried under reduced pressure.					
		The glycosides were extracted and separated by ion exchange resin chromatography and microporous resin and then dried.					
		2160 g BYHWD extract and 128 g glycosides were extracted from 100 doses of BYHWD, and the extraction rates were 19.46% and 1.15%, respectively.					
Lingguizhugan decoction	Poria cocos (Schw.) Wolf [Polyporaceae; Poria], Neolitsea cassia (L.) Kosterm. [Lauraceae; Cinnamomi ramulus], Atractylodes macrocephala Koidz. [Asteraceae; Atractylodis macrocephalae rhizoma], Glycyrrhiza uralensis Fisch. ex DC. [Fabaceae; Glycyrrhizae radix et rhizoma]	Mix in a ratio of 12:9:6:6 in sequence.	1.64 g/kg	HFD-fed mice	It reduced plasma insulin concentration, glucose tolerance, and lipids.	It restored small intestinal villus morphology and upregulated occludin levels by upregulating the relative abundance of Akkermansia, Faecalibacterium and Phascolarctobacterium.	Ning et al. (2022)
		Extraction process wasn’t provided.					

## Summarize

In conclusion, TCM can regulate intestinal tight junctions, intestinal smooth muscle motility, intestinal mucus barrier, and gut microbiota, improve intestinal hemodynamics, and alleviate systemic or general inflammatory damage and oxidative stress injury, thus exerting potential therapeutic effects on CVD. In particular, the metabolites of the gut microbiota are closely related to the development of cardiovascular diseases. By intervening in the metabolism of gut microbiota, botanical drugs reduce the level of intestinal endotoxins and attenuate the inflammatory response. It is worth noting that the effect of some of these botanical drugs on altering the body’s glycolipid metabolism and CVD risk factors is also related to the intestinal mucosal barrier. Therefore, we hypothesized that the repair of intestinal mucosal barrier damage by botanical therapy is one of the mechanisms to protect the cardiovascular system.

However, the current understanding of the relationship and mechanisms between the cardiovascular system and the intestinal mucosal barrier remains limited in contemporary research. The pharmacological and toxicological mechanisms as well as the bioavailability of Chinese medicines to the organism and the gut microbiota also need to be continuously investigated. Chinese medicines emphasize individualized treatment, giving different prescriptions according to the different stages of the disease, and most of the studies are on simple disease models, such as simple hypertension, simple heart attack, and other animal models, which do not completely simulate the real clinical situation, especially when there are multiple confounding factors such as hyperlipidemia, hyperglycemia, or renal disease in the clinical patients, which needs to be further demonstrated with a combination of disease-evidence-based modeling and a high-quality clinical randomized controlled study or a real-world studies to further prove the scientific validity. Although the TCM formula and metabolites mentioned in the article were shown to treat CVD and repair the intestinal mucosal barrier, respectively, they were not in the same study and the same model. More importantly, most of the studies only demonstrate that botanical drugs can treat both CVD and repair the intestinal mucosal barrier, but these studies are not sufficient to confirm that botanical drugs can target the intestinal tract to treat CVD, direct evidence is still scarce, and there is a need for continued research on the intermediary mediators and pathways mediated by botanical drugs and on how certain flora specifically can affect CVD.

## Figures and Tables

**Table:** 

**AB23A**	Alisol B 23-acetate
**ACAA2**	acetyl-coenzyme A acyltransferase 2
**ACE**	Angiotensin I Converting Enzyme
**AF**	atrial fibrillation
**AKT**	protein kinase B
**ANGPTL4**	Angiopoietin Like protein 4
**ApoE−/−**	Apolipoprotein E-deficient
**ARK**	Arkadia
**AS**	atherosclerosis
**BAs**	bile acids
**Bax**	Bcl-2 Associated X Protein
**BBR**	Berberine
**Bcl-2**	B-cell lymphoma-2
**BKCa**	large-conductance calcium-activated K+
**BSEP**	bile salt export pump
**BSH**	bile salt hydrolysis
**BYHWD**	Buyang Huanwu Decoction
**CA**	cholic acid
**CCl4**	Carbon tetrachloride
**CDCA**	chenodeoxycholic acid
**CHF**	chronic heart failure
**CLP**	cecal ligation and puncture
**CMs**	murine neonatal cardiomyocytes
**COX-2**	cyclooxygenase 2
**CPM**	Chinese patent medicine
**CutC**	choline trimethylamine cleavage enzyme
**CVD**	Cardiovascular disease
**CYP**	Cytochrome P450
**DAO**	diamine oxidase
**DHTS**	Dihydrotanshinone I
**DON**	deoxynivalenol
**DOX**	Doxorubicin
**FABP**	fatty acid binding protein
**FAS**	fatty acid synthase
**F/B**	Firmicutes/Bacteroidetes
**FGF15**	Fibroblast growth factor 15
**FMO**	flavin-containing monooxygenase
**FMT**	fecal microbiota transplantation
**FXR**	farnesoid X receptor
**FZD**	Fuzi decoction
**GDNF**	Glial-cell-line-derived neurotrophic factor
**GFAP**	glial fibrillary acidic protein
**GPCR**	G protein-coupled receptor
**GSK3β**	Glycosynthase kinase 3β
**GVB**	gastrointestinal vascular barrier
**HADHA**	hydroxyacyl-coenzyme A dehydrogenase
**HFD**	high fat diet
**HFHS**	high-fat/high-sugar
**HIF-1α**	Hypoxia-inducible factor 1α
**Hmox1**	hemeoxygenase
**4-HPA**	4-hydroxyphenylacetic acid
**3-HPP**	3-hydroxyphenylpropionic acid
**H/R**	hypoxia/reoxygenation
**IFN-γ**	interferon-gamma
**IgA**	immunoglobulin A
**IGF-1**	insulin-like growth factor-1
**IKKα/β**	IκB kinases
**IκB-α**	inhibitor of NF-κB
**IL**	interleukin
**I/R**	ischemia-reperfusion
**isoLCA**	isolithocholic acid
**JAK**	Janus Kinase
**JNK**	c-Jun amino-terminal kinase
**JP-2**	junctophilin-2
**LGZGD**	Lingguizhugan decoction
**LPC**	lysophosphatidylcholine
**LPS**	lipopolysaccharide
**LXR**	liver X receptor
**MAPK**	mitogen-activated protein kinase
**MCP-1**	Monocyte chemoattractant protein-1
**MDA**	mitigated malondialdehyde
**MI**	myocardial infarction
**MMP9**	matrix metalloproteinase 9
**mTOR**	mammalian target of rapamycin
**Neo**	Neohesperidin
**NF-κB**	nuclear factor kappa-B
**NGR1**	Notoginsenoside R1
**NLRP-3**	NOD-like receptor protein-3
**NOX1**	NADPH oxidases 1
**Nr1h4**	Nuclear receptor subfamily one group H member 4
**NSAIDs**	nonsteroidal anti-inflammatory drugs
**Nur77**	Nuclear hormone receptor77
**OGD**	oxygen-glucose deprivation
**ox-LDL**	oxidized low-density lipoprotein
**PAMPs**	pathogen-associated molecular patterns
**PAI-1**	plasminogen activator inhibitor-1
**PCI**	percutaneous coronary intervention
**PDH**	pyruvate dehydrogenase
**Pdk1**	pyruvate dehydrogenase kinase
**PGP9.5,**	Protein gene product 9.5
**PKK**	pyruvate kinase
**PKM2**	pyruvate kinase isoform M2
**PNS**	Panax notoginseng saponin
**PPARδ**	Peroxisome Proliferator Activated Receptorδ
**PRX3**	peroxiredoxin 3
**PXR**	pregnancy X receptor
**QL**	Qiliqiangxin
**RIMVEC**	rat intestinal microvascular endothelial cells
**ROS**	reactive oxygen species
**RSV**	Resveratrol
**SCFA**	short-chain fatty acids
**SHR**	spontaneous hypertension
**SIBO**	small intestinal bacterial overgrowth
**SMCs**	smooth muscle cells
**SREBP**	sterol regulatory element binding protein
**STAT**	signal transducer and activator of transcription
**STEMI**	st-elevated myocardial infarction
**TAK1**	transforming growth factor β-activated protein kinase 1
**TCA**	tricarboxylic acid
**TCDCCA**	taurochenodeoxycholic acid
**TCM**	Traditional Chinese Medicine
**TFEB**	transcription factor EB
**TGF-β1**	transforming growth factor
**THSWD**	Taohong Siwu Decoction
**Th17**	T-helper 17
**TLR**	Toll-like receptor
**TMA**	trimethylamine
**TMAO**	trimethylamine-N-oxide
**TNF-α**	tumor necrosis factor alpha
**TT-SR**	T-tubule-sarcoplasmic reticulum
**TXL**	Tongxinluo
**UCP2**	uncoupling protein 2
**VASP**	Vasodilator Stimulated Phosphoprotein
**Vegfa**	vascular endothelial growth factor A
**VSMCs**	vascular smooth muscle cells
**XST**	Xuesaitong
**ZO-1**	Zonula Occludens-1
